# The Cyst Nematode SPRYSEC Protein RBP-1 Elicits Gpa2- and RanGAP2-Dependent Plant Cell Death

**DOI:** 10.1371/journal.ppat.1000564

**Published:** 2009-08-28

**Authors:** Melanie Ann Sacco, Kamila Koropacka, Eric Grenier, Marianne J. Jaubert, Alexandra Blanchard, Aska Goverse, Geert Smant, Peter Moffett

**Affiliations:** 1 Boyce Thompson Institute for Plant Research, Ithaca, New York, United States of America; 2 Laboratory of Nematology, Wageningen University, Wageningen, The Netherlands; 3 INRA, Agrocampus Rennes, Univ Rennes 1, UMR1099 BiO3P (Biology of Organisms and Populations Applied to Plant Protection), Le Rheu, France; 4 Département de Biologie, Université de Sherbrooke, Sherbrooke, Québec, Canada; North Carolina State University, United States of America

## Abstract

Plant NB-LRR proteins confer robust protection against microbes and metazoan parasites by recognizing pathogen-derived avirulence (Avr) proteins that are delivered to the host cytoplasm. Microbial Avr proteins usually function as virulence factors in compatible interactions; however, little is known about the types of metazoan proteins recognized by NB-LRR proteins and their relationship with virulence. In this report, we demonstrate that the secreted protein RBP-1 from the potato cyst nematode *Globodera pallida* elicits defense responses, including cell death typical of a hypersensitive response (HR), through the NB-LRR protein Gpa2. *Gp-Rbp-1* variants from *G. pallida* populations both virulent and avirulent to *Gpa2* demonstrated a high degree of polymorphism, with positive selection detected at numerous sites. All *Gp*-RBP-1 protein variants from an avirulent population were recognized by Gpa2, whereas virulent populations possessed *Gp*-RBP-1 protein variants both recognized and non-recognized by Gpa2. Recognition of *Gp*-RBP-1 by Gpa2 correlated to a single amino acid polymorphism at position 187 in the *Gp*-RBP-1 SPRY domain. *Gp*-RBP-1 expressed from Potato virus X elicited Gpa2-mediated defenses that required Ran GTPase-activating protein 2 (RanGAP2), a protein known to interact with the Gpa2 N terminus. Tethering RanGAP2 and *Gp*-RBP-1 variants via fusion proteins resulted in an enhancement of Gpa2-mediated responses. However, activation of Gpa2 was still dependent on the recognition specificity conferred by amino acid 187 and the Gpa2 LRR domain. These results suggest a two-tiered process wherein RanGAP2 mediates an initial interaction with pathogen-delivered *Gp*-RBP-1 proteins but where the Gpa2 LRR determines which of these interactions will be productive.

## Author Summary

Biotrophic plant pathogens produce effector proteins that are delivered to the host cytoplasm where they alter defense responses and metabolism to favor pathogen colonization. In turn, plants have evolved intra-cellular proteins to recognize pathogen effector proteins, known as NB-LRR proteins, which are similar in structure to animal NOD-LRR immune receptors. While effector proteins recognized by NB-LRR proteins have been identified from many organisms, the identification of such proteins from metazoan plant parasites has presented unique challenges due to the lack of genetically tractable model species. The potato Gpa2 protein confers resistance to some isolates of the potato pale cyst nematode, *Globodera pallida*. In this report, we show that Gpa2 recognizes certain variants of the *G. pallida* protein, *Gp*-RBP-1, which is highly polymorphic both within and between populations. This recognition in turn induces defense responses, including a form of programmed cell death characteristic of plant immune receptor activation. Moreover, we show that a Gpa2-interacting protein, RanGAP2, is required for Gpa2 function and that activation of Gpa2 is enhanced when *Gp*-RBP-1 is artificially tethered to RanGAP2. Thus, our findings suggest that RanGAP2 acts as a recognition co-factor for Gpa2, and have important implications for our understanding of the mechanisms and evolution of pathogen recognition by NB-LRR proteins.

## Introduction

In plants, immune receptors encoded by disease resistance (*R*) genes confer resistance to a broad spectrum of biotrophic organisms including bacteria, fungi, oomycete, viruses, nematodes and arthropods [1]. The most numerous type of *R* genes encode intracellular proteins with nucleotide-binding (NB) and leucine-rich repeat (LRR) domains, collectively referred to as NB-LRR proteins. Two structurally different classes of NB-LRR proteins exist that encode N-terminal domains which either share homology with the Toll/Interleukin-1 Receptor (TIR) cytoplasmic domain (TIR-NB-LRR class) or have a less conserved domain with a predicted coiled-coil (CC) structure in some members (CC-NB-LRR class). Plant NB-LRR proteins show striking similarities in domain organization and predicted structure to NOD-LRR proteins, which are involved in innate immune responses in animals [2],[3]. However, unlike NOD-LRRs, which tend to recognize pathogen-associated molecular patterns (PAMPs) associated with broad classes of pathogens, NB-LRR proteins recognize proteins which are specific to a particular pathogen or pathogen isolate(s). Traditionally, these proteins are known as avirulence (Avr) proteins as they render the pathogen unable to infect a host encoding a corresponding R gene and the interaction between host and pathogen genotypes is referred to as gene-for-gene resistance. Recognition of Avr proteins by NB-LRR proteins results in the activation of defense responses that limit infection, and may lead to a characteristic form of cell death referred to as the hypersensitive response (HR).

A large number of pathogen-encoded Avr proteins from bacterial, viral, fungal and oomycete plant pathogens have been identified that elicit NB-LRR-mediated resistance [1]. Some Avr-encoding genes show hallmarks of selection pressure, manifested as sequence diversification or gene deletions that have allowed escape from host detection suggesting that pathogens are subject to strong selective pressure to avoid recognition by components of the plant innate immune system [4]. Avr proteins recognized by NB-LRR proteins show little structural commonality except that they are either synthesized in (in the case of viruses), or delivered to the host cytoplasm by various pathogen protein delivery systems. In the absence of a corresponding R protein, most Avr proteins are thought to act as effector proteins to enhance pathogen virulence. As such, *R* gene mediated resistance is often referred to as effector-triggered immunity (ETI) [5]. It has been suggested that NB-LRR proteins have evolved to “guard” cellular targets of effectors by responding to their alteration [6]. Alternatively, the decoy model suggests that NB-LRR proteins might recognize effectors not by interacting with virulence targets *per se*, but with proteins that simply resemble effector targets [7]. *Avr* genes from microbial pathogens have traditionally been identified by genetic approaches. Genetic identification of Avr genes from metazoan parasites has been challenging however, owing to the complexity of their genomes and life cycles, and a paucity of genetically tractable model organisms. This hindrance is particularly acute for plant parasitic nematodes, necessitating alternate approaches to identifying Avr candidates.

Cyst nematodes of the genus *Globodera* are obligate plant parasites, spending the majority of their life cycle within roots. These nematodes develop an intimate relationship with their host *via* the induction of a complex feeding site structure, known as the syncytium, in the vascular cylinder of the potato roots. Cyst nematodes produce an assortment of parasitism proteins in order to infect plants, which in principle can be thought of as being analogous to effector proteins of microbial pathogens [8],[9]. These proteins are synthesized in the oesophageal glands (two sub-ventral and one dorsal) and some of these are injected into the host cytoplasm using a specialized structure called the oral stylet. Both host range specificity and suppression of host plant resistance are thought to be controlled by nematode effector proteins [10]. Many putative nematode effector proteins have been identified by virtue of their possession of a protein sorting signal for extracellular secretion and expression in the esophageal gland [8]. In theory, these proteins also have the potential to be recognized by NB-LRR proteins. To date, however, there are no unambiguous reports of nematode effector proteins that also elicit defense responses by specific NB-LRR proteins.

Use of plant nematode resistance genes is an effective and environmentally safe method for managing these parasites. Four nematode *R* genes encoding NB-LRR proteins have been identified in *Solanaceous* species [11]. *Gpa2* is a potato gene that encodes a CC-NB-LRR protein and confers resistance against two field populations (D383 and D372) of *G. pallida* [12],[13],[14]. In *Gpa2*-expressing potatoes, nematodes penetrate roots, start the initiation of their feeding site and become sedentary. However, the tissue surrounding the developing feeding site subsequently becomes necrotic and collapses, suggesting the elicitation of an HR. *Gpa2* is closely related to the *Rx* and *Rx2* genes, which confer resistance to Potato Virus X (PVX), through recognition of the viral coat protein (CP). Rx function is dependent on Ran GTPase-activating protein 2 (RanGAP2), a protein shown to interact with the N-terminal CC domains of Rx, Rx2 and Gpa2 [15],[16]. Domain swap experiments have shown that the N-terminal halves of the Rx and Gpa2 proteins are interchangeable for mediating HR responses in response to the PVX CP whereas the LRR domain determines recognition specificity [17].

In this report, we used a candidate gene approach to test the possibility that the *G. pallida* RBP-1 protein may possess avirulence activity towards Gpa2. *Gp*-RBP-1 possesses a secretion signal peptide, is expressed in the *G. pallida* dorsal esophageal gland, and is most closely related to a family of proteins from *G. rostochiensis*, the secreted SP1a and RYanodine receptor (SPRY) domain (SPRYSEC) proteins, which have been shown to be present in stylet secretions [18],[19],[20]. RBP-1 and SPRYSEC proteins possess a SPRY domain that most closely resembles the Ran GTPase-associated protein, Ran-Binding Protein in the Microtubule-organizing center (RanBPM) [19], a multi-domain protein conserved in most eukaryotes [21],[22]. The SPRY domain of *Gp*-RBP-1 is part of a B30.2 domain, an extended domain structure comprising PRY and SPRY subunits [18]. We show that *Gp*-RBP-1 variants are highly variable within and between populations and appear to be under positive selection, with maintenance of avirulent (recognized by Gpa2) *Gp*-RBP-1 variants in populations not controlled by Gpa2. We also present data suggesting that recognition of *Gp*-RBP-1 by Gpa2 is mediated by an initial interaction with RanGAP2 but that the Gpa2 LRR domain determines which *Gp*-RBP-1 variants elicit activation of Gpa2. Implications for mechanisms of recognition and selection pressures on nematode effector proteins are discussed.

## Results

### Identification of a *G. pallida* AvrGpa2 candidate

The NB-LRR protein Gpa2 has previously been shown to interact with the Ran GTPase activating protein RanGAP2, which in turn is predicted to interact with Ran GTPase as part of its normal cellular function in nucleocytoplasmic trafficking and mitosis [15],[23]. RBP-1 shares homology to the SPRY domain of RanBPM, which has also been annotated as being a Ran GTPase-binding protein. Both the guard and the decoy models predict that NB-LRR proteins recognize Avr proteins through interactions with a common protein partner. Thus, given the predicted potential convergence of Gpa2 and RBP-1 on Ran GTPase or affiliated proteins, we reasoned that Gpa2 might recognize an RBP-1 homologue, *Gp*-RBP-1, from *G. pallida* [18].

**Figure 1:**
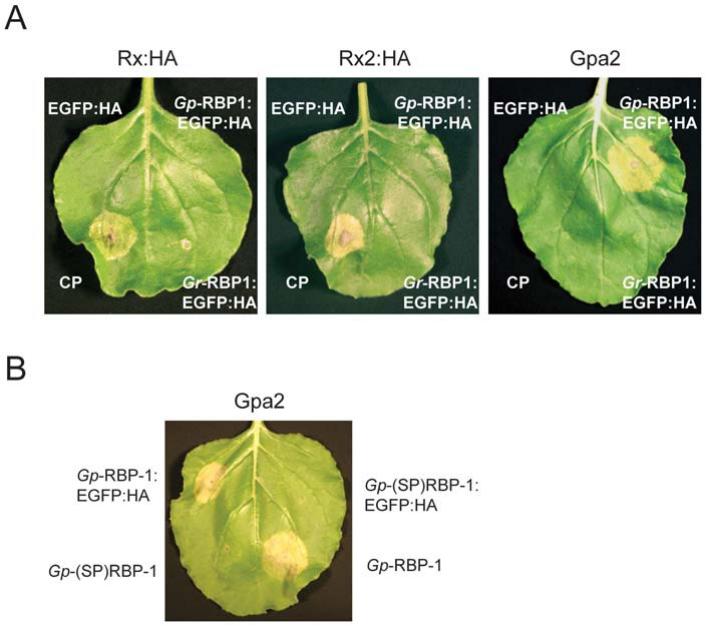
*Gp*-RBP-1 induces a Gpa2-mediated HR in *Nicotiana benthamiana* leaves. (A) HA-tagged Rx and Rx2, or untagged Gpa2 driven by the *Rx* promoter were transiently expressed via agro-expression in wild-type *N. benthamiana* leaves together with 35S promoter-driven PVX CP or a *G. pallida* RBP-1 protein cloned from the population Chavornay (Chav-1) fused to a C-terminal EGFP fusion and epitope tag (EGFP:HA). EGFP:HA and a *G. rostochiensis* RBP-1: EGFP:HA fusion were included as controls. HRs were observed within 2 to 3 days of ago-expression. (B) Tagged and untagged versions of *Gp*-RBP-1 were also tested that included the 23 amino acid secretion signal peptide (SP) from the predicted full-length *Gp*-RBP-1 protein [*Gp*-(SP)RBP-1 and *Gp*-(SP)RBP-1:EGFP:HA]. HRs were observed within 2 to 3 days of ago-expression.

One of the hallmarks of Avr recognition by NB-LRR proteins is the induction of an HR when both proteins are present in the same cell. As such, we tested whether *Gp*-RBP-1 could induce a Gpa2-dependent HR in a transient expression assay. A *Gp-Rbp-1* cDNA derived from *G. pallida* pathotype (Pa-) 2/3 population Chavornay was cloned into the binary vector pBIN61 under control of the cauliflower mosaic virus 35S promoter as a C-terminal HA-tagged EGFP fusion (*Gp*-RBP-1:EGFP:HA), but lacking its secretion signal peptide. This protein was transiently co-expressed with *Gpa2* driven by the *Rx* genomic promoter using *Agrobacterium*-mediated expression (agroinfiltration) in *N. benthamiana* leaves. *Gp*-RBP-1:EGFP:HA elicited an HR in the infiltration patch within three to four days (Figure 1A). An equivalent fusion protein with a SPRYSEC homolog from *Globodera rostochiensis* (*Gr*-RBP-1:EGFP:HA), which shares 43.7% amino acid similarity [18],[20], did not elicit Gpa2-mediated HR, nor did the control proteins EGFP:HA or the coat protein (CP) from potato virus X (PVX). Rx and Rx2 were also tested for recognition of *Gp*-RBP-1:EGFP:HA, but both NB-LRR proteins showed strict specificity for the PVX CP (Figure 1A). It is predicted that the native secretion signal peptide of *Gp*-RBP-1 would be required for secretion from the nematode esophageal gland cells, whereupon it would be cleaved off and the mature protein delivered to the host cytoplasm via the nematode stylet. The same signal peptide would also be predicted to direct co-translational translocation to the ER for secretion from the plant cell, preventing cytoplasmic accumulation of the native protein. Indeed, as predicted, no HR was induced when the native secretion signal peptide sequence was retained in *Gp*-RBP-1 (Figure 1B), consistent with it being recognized by Gpa2 intra-cellularly. Untagged *Gp*-RBP-1 also induced a Gpa2-specific HR, indicating that recognition by Gpa2 was not an artifact of the EGFP fusion protein (Figure 1B). These results indicate that the Gpa2 protein has the capacity to recognize *Gp*-RBP-1, and in turn induce a typical HR.

### *Gp-Rbp-1* is highly polymorphic and subject to positive selection

**Figure 2:**
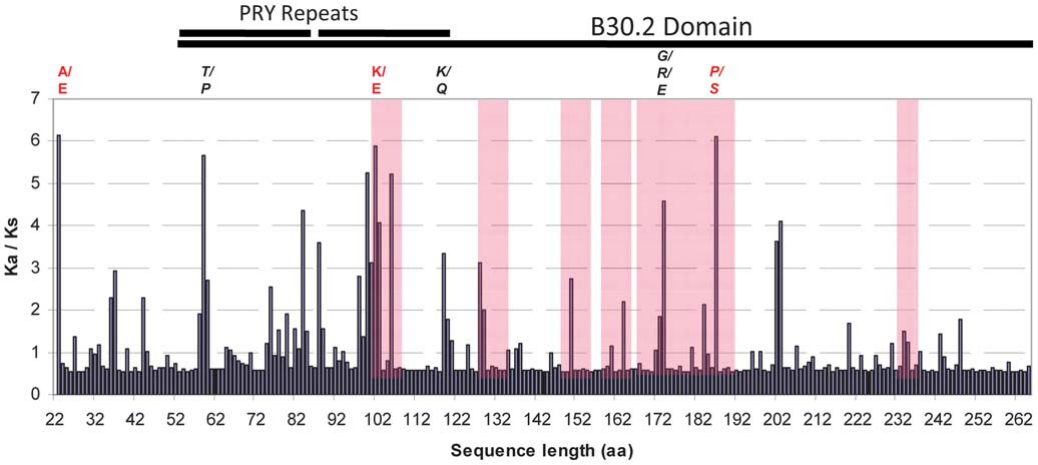
Distribution of the Ka/Ks ratio along the RBP-1 amino acid sequence. Analyses were conducted using the codeml module of PAML on the full data set of *G. pallida* and *G. mexicana* sequences. Amino acid variants found to be subjected to positive selection with posterior probability >95% (Table S1A) are indicated in red above each site. Amino acid variants found to be subjected to positive selection in PAML and at least one other method (Table S1B) are indicated in italic. Sequence portions corresponding to the SPRYSEC extended loops in the B30.2 protein structure are highlighted in pink. The entire B30.2 domain is indicated by a bar above the graph, with the region containing the duplicated PRY domains indicated by double bars.

We analyzed a number of additional sequences from several *G. pallida* populations including some from the native range of this parasite (Peru), as well as two sequences from the very closely related species *G. mexicana* (Figure S1). RBP-1 homologues possess an N-terminal secretion signal peptide (SP) followed by a B30.2 domain which is comprised of juxtaposed PRY and SPRY domains [18],[24]. *Gp*-RBP-1 sequences differed by single amino acid residue polymorphisms, insertions and deletions, but were all structurally similar, with an additional, near-perfect repeat of the PRY domain immediately N-terminal to the B30.2 domain, whereas all *G. mexicana* sequences possessed only a single PRY domain (Figures 2 and S1).

To determine whether positive selection pressure could be detected in this dataset, we applied the site-specific likelihood models implemented in the CODEML program (M1 vs M2 and M7 vs M8) of the PAML (phylogenetic analysis by maximum likelihood) package [25],[26]. These models assume variable selective pressures among sites but no variation among branches in the phylogeny. The PAML M8 and M2 models of positive selection appeared to be significantly (p<0.001) better adapted to the data set (Table S1A) showing that RBP-1 has indeed been subjected to positive selection at numerous sites along the protein sequence (Figure 2). To determine among the PAML detected sites those supported by other methods, we carried out complementary evolutionary analyses using the SLAC, REL and FEL maximum likelihood methods implemented in the HYPHY program [26]. Only four sites were supported by at least two different methods (Table S1B) and only residue 187 was detected as being under positive selection by all four methods with strong statistical values. Residues 187, 174 and 102 localize to predicted extended loops that shape the surface A of the SPRY domain based on the comparison to SPRYSEC-19 [21] (Figure 2).

### *Gp*-RBP-1 Variants from both Avirulent and Virulent Populations Elicit Gpa2

**Figure 3:**
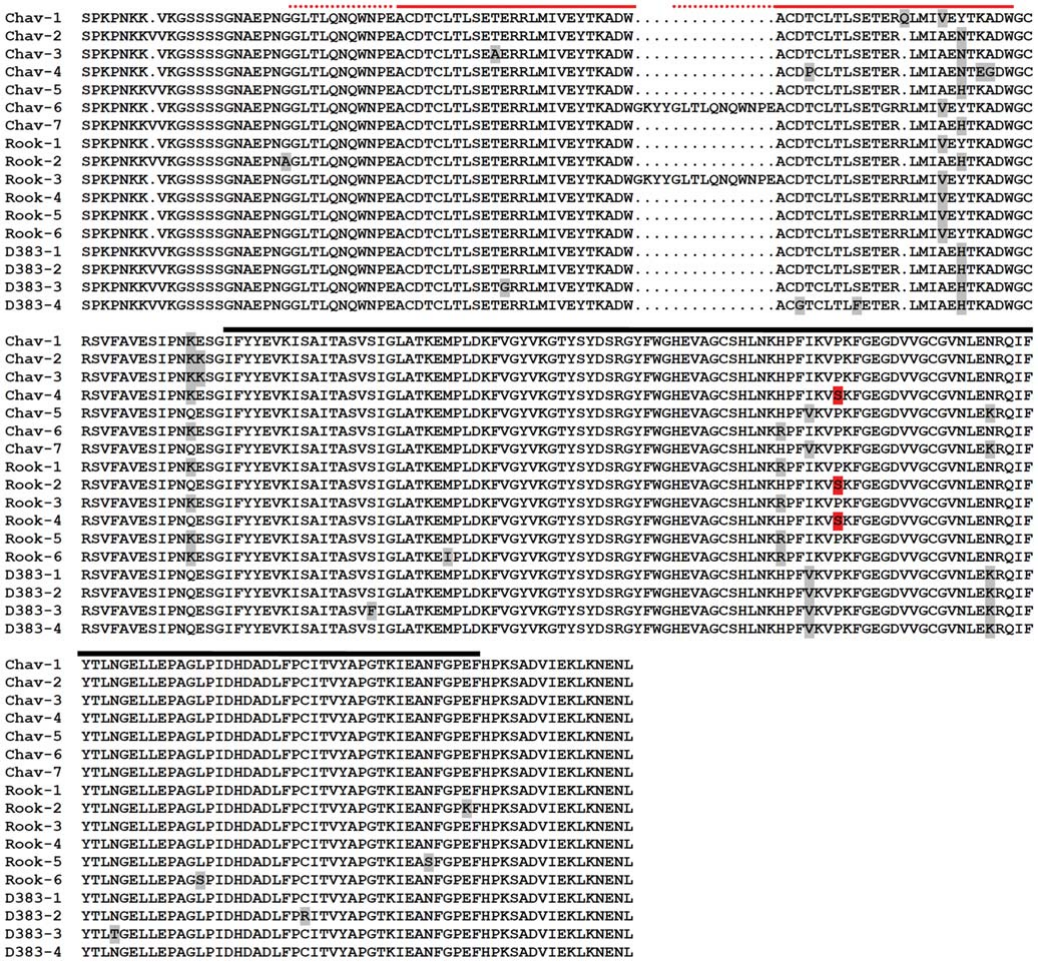
Analysis of *Gp*-RBP-1 variants from virulent and avirulent populations. Alignment of deduced *Gp*-RBP-1 proteins encoded by cDNA sequences cloned from *G. pallida* populations D383 (avirulent; pathotype Pa-2), Rookmaker (virulent; Pa-3) and Chavornay (virulent; Pa-2/3). Variant residues are indicated with shading, with the critical proline/serine polymorphism indicated in red. PRY domain repeats are indicated by a red bar over the alignment, with the dashed segment of the bar corresponding to an extension of the repeat in two of the variants. The SPRY homology domain is overscored by the black bar.

The *Gpa2* gene restricts only a limited subset of *G. pallida* populations [14]. However, the possibility that virulent and avirulent individuals might co-exist within virulent populations has not been examined. We focused on the pathotype 2 (Pa-2) population D383, which is avirulent on *Gpa2* plants, and the virulent pathotype 3 (Pa-3) population Rookmaker [27], as well as Chavornay (Pa-2/3), to seek correlations between recognition by Gpa2 and the polymorphisms within and between these populations. Of a total of 76 sequences derived from RT-PCR from multiple individuals from either D383 or Rookmaker populations, we obtained four different sequences from D383 (D383-1, 37 times; D383-2, twice; D383-3, once; D383-4, once) and six from Rookmaker (Rook-1, 18 times; Rook-2, 8 times; Rook-3, 4 times; Rook-4, twice; Rook-5, twice; Rook-6, once). The *Gp*-RBP-1 sequences deduced from these populations showed a number of insertion/deletion polymorphisms and amino acid substitutions (Figure 3). Most notably, Chav-6 and Rook-3 showed a 15 aa insertion that is highly similar in length and sequence to that encoded by *Gp-Rbp-1* intron 3 (44 bp in length) [18]. Thus, some *Gp*-RBP-1 isoforms may be expressed by alternative splicing although the possibility that these clones represent different alleles of the same gene or different gene copies cannot be discounted. Indeed, since these sequences were identified from a population of individuals, we cannot definitively conclude whether all the sequences we have analyzed derive from different alleles of the same gene or from different gene copies. However, the diversity seen herein is a characteristic often seen in pathogen Avr genes [28],[29].

**Figure 4:**
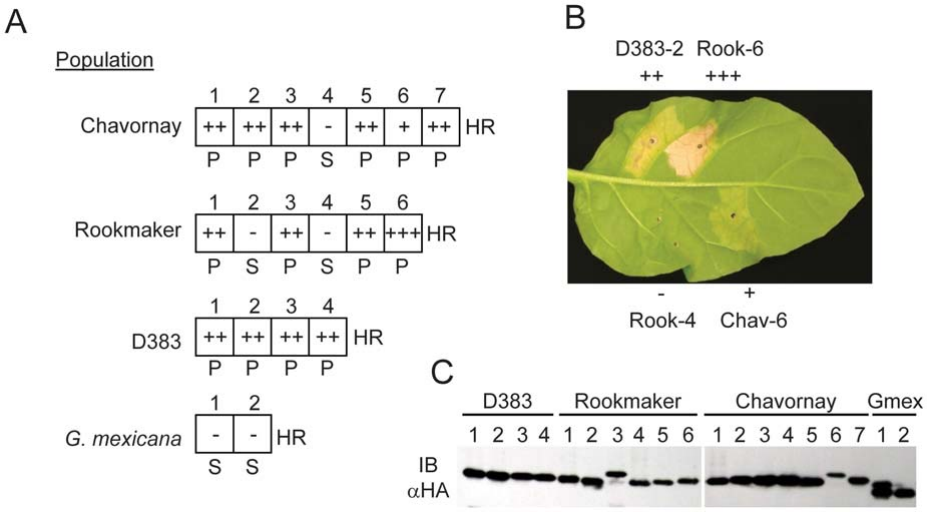
Recognition of *Gp*-RBP-1 by Gpa2 corresponds to avirulence, but not virulence in *G. pallida* populations. (A) *Gp*-RBP-1 variants (shown in Figure 3) cloned into pBIN61 as HA-tagged proteins under control by the CMV 35S promoter were transiently expressed via agro-infiltration on GPAII::Gpa2 transgenic tobacco. The responses in the infiltrated patches were scored visually with a complete lack of response scored as (-). Positive HR responses were scored as follows: complete collapse and rapid desiccation of the infiltration patch within 2 days (+++), complete collapse of the infiltration patch by 3 days post-infiltration (++), or slow and incomplete collapse with residual live cells (+). HR phenotypes representative of the scale used herein are shown (B), as photographed seven days after infiltration. The presence of either a proline (P) or serine (S) residue at the position corresponding to Rook-1 residue 187 is indicated. (C) Immunoblot with horse radish peroxidase-conjugated anti-HA antibody demonstrating relative protein levels of transiently expressed RBP-1 proteins.

To test for recognition by Gpa2, the open reading frames, minus the SP, of the seventeen different *Gp*-RBP-1 variants identified from the D383, Rookmaker and Chavornay populations were cloned in frame with a C-terminal hemagglutinin (HA) epitope tag. All clones from the avirulent population D383 induced a Gpa2-specific HR on *Gpa2*-transgenic *N. tabacum* (tobacco; Figure 4A). Several *Gp*-RBP-1 variants from Chavornay and Rookmaker were also recognized by Gpa2, although some differences in HR strength were consistently observed (Figure 4A). Three variants (Chav-4, Rook-2 and Rook-4) failed to elicit a Gpa2-dependent HR despite the detection of similar protein levels of all variants by immunoblotting (Figure 4C). We also tested two RBP-1 variants (Gmex-1 and Gmex-2) from *G. mexicana*, which share high degrees of amino acid sequence similarity with *Gp*-RBP-1 proteins but encode only a single PRY domain (Figure S1). Neither of these *Gm*-RBP-1 proteins elicited a Gpa2-dependent HR (Figure 4).

### A Single Residue Determines Gpa2 Recognition of *Gp*-RBP-1

**Figure 5:**
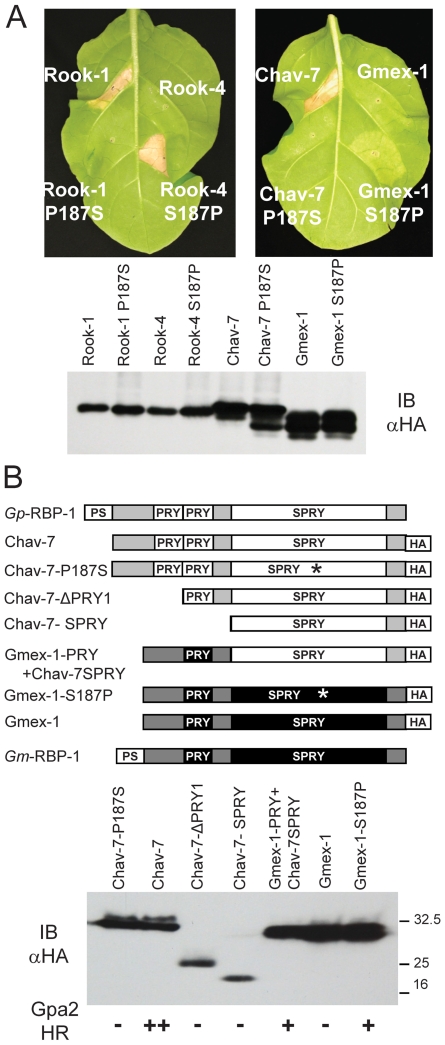
A single residue in the *Gp*-RBP-1 SPRY domain is a key determinant of Gpa2 recognition. (A) Proline 187 of Rook-1 and Chav-7 was substituted for serine, and serine 187 of Rook-4 and Gmex-1 was substituted for proline. The resulting RBP-1:HA proteins were transiently expressed in *Gpa2* tobacco leaves. Note that Rook-4 S187P induced an HR of a strength equivalent to those elicited by Rook-1 and Chav-7 (+++ as per Figure 4B), whereas Gmex-1 S187P induced a much weaker response (+ as per Figure 4B). RBP-1:HA variants were also expressed in wild-type tobacco and protein extracts were subjected to anti-HA immunoblotting (IB) to determine protein expression levels (lower panel). (B) Deletions of, and fusions between, *G. pallida* Chav-7 and *G. mexicana* Gmex-1 RBP-1:HA are represented schematically. Individual proteins were expressed in wild-type tobacco and protein extracts were subjected to anti-HA immunoblotting to determine protein expression levels (lower panel). Individual proteins were scored for their ability to induce an HR on *Gpa2*-transgenic tobacco as per the scale in Figure 4B.

Despite numerous polymorphisms in *Gp*-RBP-1 variants, only a proline/serine polymorphism at position 187, relative to the reference full-length Guic-3 sequence (Figure S1), correlated with recognition by Gpa2 (Figures 3 and 4A). This residue was also shown to be under positive selection (Figure 2 and Table S1). To test the importance of residue 187 in recognition by Gpa2, we substituted serine and proline codons at position 187 in Rook-1, Rook-4, Chav-7, and Gmex-1. The substitution of proline 187 to serine in Rook-1 and Chav-7 abolished recognition by Gpa2, whereas substitution of serine 187 to proline in Rook-4 and Gmex-1 resulted in a gain of recognition by Gpa2, although the Gmex-1 S166P protein elicited only a very weak HR (Figure 5A). Altered recognition of amino acid 187 substitution proteins did not result from large changes in *Gp*-RBP-1 protein accumulation, as demonstrated by immunoblot detection of wild-type and mutant constructs (Figure 5A). An additional degradation product was seen for the Chav-7 P187S construct, but levels of the intact protein resembled that of the wild-type Chav-7 *Gp*-RBP-1; moreover, this degradation product was not observed for the equivalent Rook-1 P187S construct, suggesting it was unlikely that the degradation product affects recognition. These observations are consistent with an absolute requirement for a proline residue at position 187, but suggest that other regions of the protein likely modulate the potential for recognition by Gpa2.

To explore further the role of the structurally variable RBP-1 N terminus in recognition by Gpa2, we tested constructs of Chav-7 with serial deletions of its PRY sequences, and exchanged the Gmex-1 SPRY domain for that from Chav-7 (Figure 5B). Chav-7 deletants lost their ability to elicit Gpa2, however, immunoblot detection demonstrated that these proteins accumulated to lower levels, suggesting that the deletions may destabilize the protein. On the other hand, the chimeric protein comprising the single PRY domain from Gmex-1 and the Chav-7 SPRY domain was recognized by Gpa2, albeit, to a lesser degree (Figure 5B). This result indicates that an intact N-terminus is required for recognition of *Gp*-RBP-1 by Gpa2, and that variation in this region of the protein can influence the strength of recognition by Gpa2.

### RanGAP2 is required for HR Induced through Gpa2

**Figure 6:**
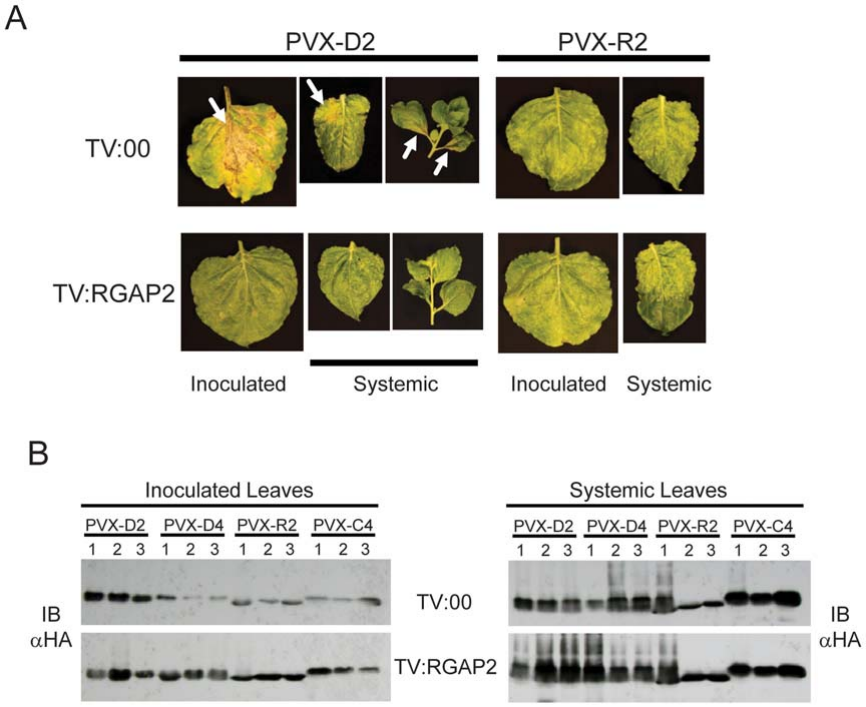
Gpa2-mediated responses to PVX-RBP-1:HA requires RanGAP2. PVX vectors were generated to express two avirulent versions (D383-2 and D383-4) of *Gp*-RBP-1:HA (PVX-D2 and PVX-D4) as well as two virulent (Rook-2 and Chav-4) variants (PVX-R2 and PVX-C4). (A) Virus saps containing recombinant viruses were rub-inoculated onto *Gpa2*-transgenic *N. benthamiana* that had previously been infected with the empty TRV VIGS (TV:00) vector or TRV:RGAP2. Phenotypes from a representative experiment are shown for PVX-D2 and PVX-R2, photographed two weeks after PVX inoculation. Virus spread to systemic tissues was observed either by the development of systemic lesions and necrosis (PVX-D2 and PVX-D4) or PVX symptoms typical of infected wild-type plants (PVX-R2 and PVX-C4). Necrosis on local and systemic leaves is indicated by arrows. (B) Protein extracts taken from inoculated and systemic leaves of *Gpa2*-transgenic *N. benthamiana* plants, infected as in (A), were subjected to anti-HA immunoblotting (IB) to detect *Gp*-RBP-1:HA accumulation.

Previously, the RanGAP2 protein was shown to interact with the N-terminal CC domains of both Rx and Gpa2, and to be required for Rx-induced responses to PVX [15],[16]. A lack of workable reverse genetic approaches precluded an investigation of the requirement for RanGAP2 in the potato-nematode interaction. Therefore, to test the requirement for RanGAP2 in Gpa2-mediated responses, we generated transgenic *N. benthamiana* expressing Gpa2 from the *Rx* genomic promoter as well as PVX derivatives expressing Gpa2-eliciting (D383-2 or D383-4; PVX-D2 and PVX-D4) or non-eliciting (Rook-2 or Chav-4; PVX-R2 and PVX-C4) versions of *Gp*-RBP-1. RanGAP2 expression was silenced by virus-induced gene silencing (VIGS) using a tobacco rattle virus (TRV) vector [15]. As a control, plants were inoculated with the empty TRV vector (TV:00). Rub-inoculation of TV:00-infected plants with PVX expressing either PVX-D2 or PVX-D4 resulted in the presentation of HR-type lesions in the inoculated leaves (Figure 6A). However, resistance responses induced by Gpa2 failed to prevent systemic spread of the recombinant viruses, resulting in a spreading systemic HR (SHR; Figure 6A). Although this response differs from the Rx-mediated response to most PVX strains [12] it resembles the response seen in *Rx* transgenic *N. benthamiana* infected with a strain of PVX weakly recognized by Rx [30]. Indeed, SHR-type responses are commonly seen in interactions between *R* genes that are not able to fully contain virus infection due to weak recognition [31]. In contrast, PVX-R2 and PVX-C4 did not induce HR lesions or SHR (Figure 6A). Silencing of RanGAP2 abrogated both the induction of local HR and SHR by PVX-D2 and PVX-D4, demonstrating a requirement for RanGAP2 in Gpa2 function (Figure 6A).

To complement our VIGS experiments, we also used a dominant-negative approach to block RanGAP2 function in Gpa2-mediated responses. Plant RanGAP proteins possess a plant-specific N-terminal WPP domain that includes a three amino acid signature motif (WPP) shown to be essential for concentrating RanGAP1 protein to the cytoplasmic side of the nuclear envelope as well as the cell division plane [23],[32]. The Rx CC domain interacts with RanGAP2 through the WPP domain [16] as does the Gpa2 CC domain (Figure S2). We fused the WPP of RanGAP2 to EGFP:HA (WPP:EGFP:HA) and used this construct to stably transform *N. benthamiana*, with control transgenic lines generated to express EGFP:HA. Over-expression of WPP:EGFP:HA completely blocked the HR elicited by transient expression of Gpa2 plus *Gp*-RBP-1:EGFP:HA (Figure S2B). However, it had no effect on the CP-dependent HR elicited by Rx or by Pto plus AvrPto (Figure S2B). Although interference by WPP:EGFP:HA appeared to be specific to Gpa2, we do not rule out the possibility that residual endogenous RanGAP2 activity may be sufficient for Rx function, which normally mediates a more rapid and stronger HR than Gpa2.

### Artificial tethering of RanGAP2 and *Gp*-RBP-1 enhances Gpa2-mediated HR

A number of proteins that interact with the N termini of NB-LRR proteins mediate Avr recognition by their cognate NB-LRR partner [33],[34],[35],[36] and we have previously suggested that RanGAP2 may play a similar role with by Rx and Gpa2 [15]. However, we have been unable to consistently show a direct interaction between *Gp*-RBP-1 and potato RanGAP2 by yeast two-hybrid or co-immunoprecipitation (M.A.S. and P.M., unpublished data). In an attempt to demonstrate in situ interactions, we employed the bimolecular fluorescence complementation (BiFC) technique using split YFP fragments [37]. Constructs were generated to fuse either the N-terminal or C-terminal YFP fragments, plus a FLAG epitope tag, to the C-termini of proteins of interest (nYF and cYF).

**Figure 7:**
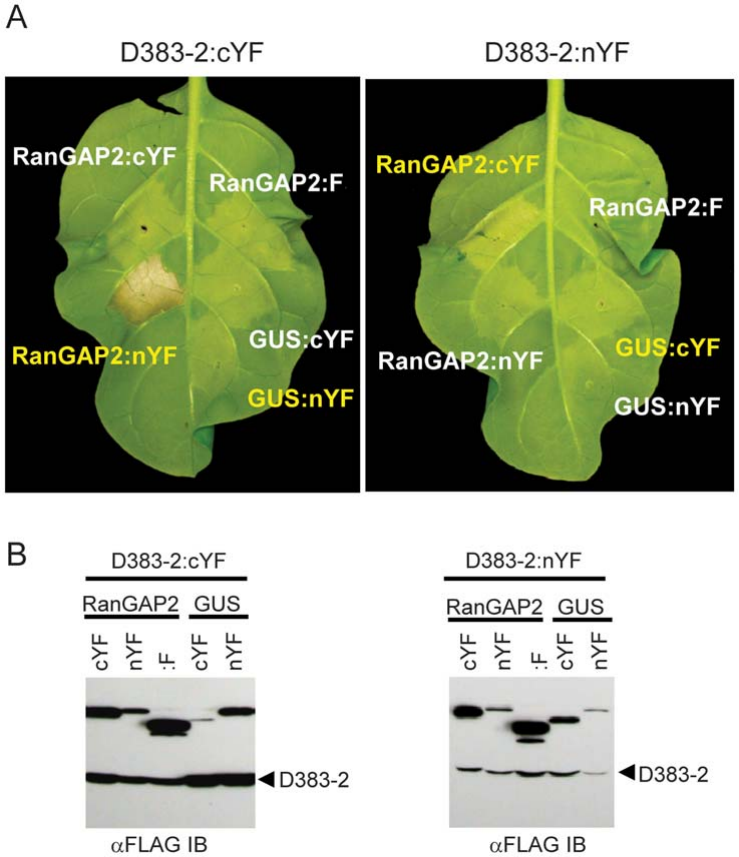
Tethering of RanGAP2 and *Gp*-RBP-1 enhances Gpa2-mediated HR. (A) The open reading frames of RanGAP2, *Gp*-RBP-1 clone D383-2 and GUS were fused at their C termini to either the C-terminal or N-terminal fragments of YFP:FLAG (cYF and nYF, respectively). D383-2:cYF and D383-2:nYF were co-expressed, by agro-infiltration, in *Gpa2*-transgenic tobacco together with both complementing fusion proteins (yellow) and non-complementing YFP fusion proteins (white) as indicated (top panel). RanGAP2 with only a C-terminal FLAG tag (RanGAP2:F) was included as an additional non-complementing control. (B) Fusion proteins were also expressed in wild-type tobacco and protein extracts were subjected to anti-FLAG immunoblotting (IB) to confirm that activation in the combinations with complementing YFP fragments did not correlate with the highest RanGAP2 levels.

BiFC fusion proteins were first tested for functionality in HR assays. Although the *Gp*-RBP-1 (D383-2) protein elicits a Gpa2-dependent HR within three days of agroinfiltration (++, Figure 4A), fusion of *Gp*-RBP-1 (D383-2) with the YFP fragments (D383-2:nYF and D383-2:cYF) resulted in a much weaker elicitation of Gpa2-mediated HR (+ as per the scale in Figure 4B). However, we observed a strong HR (+++ as per Figure 4B) upon co-expression D383-2:cYF with RanGAP2 fused to the nYFP fragment (RanGAP2:nYF) in *Gpa2*-transgenic tobacco leaves (Figure 7A). A similar, albeit less pronounced, HR enhancement was seen with the reciprocal combinations of complementing YFP fragments, D383-2:nYF and RanGAP2:cYF (Figure 7A). The weaker response seen with the D383-2:nYF fusion in the absence of complementation, however appears to correlate with its relatively lower level of accumulation (Figure 7B). Comparison of protein expression levels of RanGAP2:cYF, RanGAP2:nYF and RanGAP2 with only a FLAG tag (RanGAP2:F) showed that HR enhancement correlated with the presence of complementing YFP fragments, and not protein expression levels (Figure 7B). As an additional control, D383-2:nYF and D383-2:cYF were co-expressed with GUS YFP fragment fusions, GUS:nYF and GUS:cYF, neither of which showed any effect on enhancing the Gpa2-mediated HR (Figure 7A).

The reconstitution of YFP fragments is irreversible [38]. Indeed, we find that all combinations of HA- or FLAG-tagged nYFP and cYFP fusion proteins that we have tested interact and can be efficiently co-immunoprecipitated (Figure S4, MAS and MJJ unpublished data). Since the control protein GUS also interacted with all proteins tested in this assay (Figure S4) split YFP reconstitution appears to be highly promiscuous in plants as long as the cognate fusion proteins are stably expressed. Nevertheless, we reasoned that if the recognition by Gpa2 is mediated by a weak or transient interaction between RanGAP2 and *Gp*-RBP-1, then strengthening such an interaction would strengthen the degree of Gpa2 activation. To test the specificity of this phenomenon we introduced *Gp*-RBP-1 (Rook-4), which is not recognize by Gpa2 (Figure 4A) into the split YFP assay with RanGAP2. Although YFP complementation allowed these two proteins to interact physically, it did not result in a gain of recognition of *Gp*-RBP-1 (Rook-4) by Gpa2 (Figure S3A). Moreover, complementing pairs of *Gp*-RBP-1 and RanGAP2 did not activate the Rx protein (Figure S5). These results suggest that the artificial tethering of *Gp*-RBP-1 proteins to RanGAP2 mimics and enhances an interaction that normally occurs between these proteins, but that interaction alone is not sufficient to activate the associated NB-LRR protein. Thus, although RanGAP2 is involved in an initial phase of Avr interaction, recognition specificity is nonetheless determined by the NB-LRR protein.

## Discussion

Given a lack of consistent reverse genetics tools for cyst nematodes, we have used functional assays to demonstrate avirulence activity of *Gp*-RBP-1 as defined by the ability of a protein to elicit defense responses by a specific R protein. The presence of matching R and Avr proteins is generally sufficient to induce resistance response, the most obvious being the HR. Our data show that specific *Gp*-RBP-1 variants induce an HR only in the presence of Gpa2 but not Rx or Rx2 (Figures 1 and 4). Thus, by definition, these proteins possess Gpa2 avirulence activity and at a functional level represent a gene-for-gene relationship. Furthermore, these same *Gp*-RBP-1 proteins elicit resistance responses, manifested as systemic HR, when expressed from PVX (Figure 6). The fact that Gpa2 does not fully restrict these recombinant viruses is likely due to the relatively rapid movement of PVX from infected cells, similar to what is seen with versions of PVX that are weakly recognized by Rx [30]. This is consistent with the fact that most *Gp*-RBP-1 variants induced a Gpa2-mediated HR only after three days (Figure 4), whereas the Rx/CP-mediated HR occurs within 24 hours (P. Moffett, unpublished observations). Furthermore, even on *Gpa2* potato plants avirulent *G. pallida* induce an HR only after 7–9 days, (K. Koropacka, unpublished observations) suggesting that the Gpa2 response is relatively weak, possibly due to an inherently weak recognition of Avr proteins. Since the nematode does not move from its initial feeding site, this slow response may be sufficient for nematode resistance whereas it results in SHR in the case of a viral infection.

While *Gp*-RBP-1 alleles displayed many polymorphisms, recognition by Gpa2 could be attributed to a single proline/serine polymorphism in the SPRY domain (Figure 5). However, although a proline at position 187 appears to be absolutely necessary for Gpa2 activation, variations at other sites likely modified the strength of HR induced through Gpa2 and a nearly-intact protein is required for Avr activity (Figures 4 and 5). We only recovered avirulent variants of *Gp*-RBP-1 from the avirulent population D383, consistent with a role for this nematode protein in eliciting Gpa2-mediated resistance. However, both Gpa2-recognized and non-recognized variants of *Gp*-RBP-1 were isolated from two *G. pallida* populations (Rookmaker and Chavornay) virulent to *Gpa2*. It is possible that these versions of *Gp*-RBP-1 are not expressed although this seems unlikely as their isolation depended on the expression of their mRNAs. These data suggest rather, that field populations contain both virulent and avirulent individuals, consistent with the fact that *Gpa2* has not been effective in the field.

On the other hand, it is possible that *Gp*-RBP-1 is not the sole determinant of avirulence among different *G. pallida* populations. A recent report showed that a key gene from the root-knot nematode *Meloidogyne incognita* determining avirulence to the tomato *Mi-1* gene, designated *Cg-1*, could encode an RNA that regulates avirulence. The longest open reading frame (ORF) in *Cg-1* has the capacity to encode a polypeptide of only 32 amino acids without the appearance of signal sequence [39]. It is unlikely that a product of the *Cg-1* gene ultimately elicits the Mi-1 protein and yet silencing of *Cg-1* in the nematode compromised resistance conferred by the *Mi-1* gene. Thus, avirulence as defined genetically, may not correlate absolutely with the possession of a gene encoding avirulence activity, as defined by the elicitation of an R protein by a pathogen-derived molecule. Indeed, this concept is not without precedent. For example, in *Pseudomonas syringae* the effector protein AvrRpt2 interferes with recognition of AvrRpm1 by the NB-LRR protein RPM1, while the effectors VirPphA and AvrPtoB are able to suppress the HR responses induced by co-delivered Avr proteins [40],[41],[42]. Suppression of Avr recognition by NB-LRR proteins can be highly specific as in the case of the flax TIR-NB-LRR L6 and L7 proteins which recognize the same versions of flax rust AvrL567 proteins but are differentially suppressed by the presence of the flax rust inhibitor (*I*) gene [28],[43],[44]. Furthermore the oomycete protein ATR13^Emco5^ confers avirulence toward the Arabidopsis *RPP13* gene in the ecotype Nd-0 but not ecotype Ws-0, despite the ability of RPP13 to recognize bacterially-delivered ATR13^Emco5^ in both ecotypes [45]. This is reminiscent of the ability of the *Pseudomonas syringae* protein AvrPphC to suppress recognition of AvrPphF, but only in certain bean cultivars [46]. Thus it would appear that the ultimate outcome of the interaction between a given pair of Avr and R proteins can be influenced by additional factors determined by the genotypes of both the pathogen and the host. Only forms of *Gp*-RBP-1 avirulent to Gpa2 were found in population D383 suggesting that this is a prerequisite for Gpa2-mediated resistance. However, the identification of forms of *Gp*-RBP-1 avirulent to Gpa2 in the Rookmaker population might suggest that additional factors present in this population may act epistatically to *Gp*-RBP-1, either suppressing recognition of *Gp*-RBP-1 by Gpa2 or the ensuing defense responses.

Although this report does not fully address the extent of variability of *Gp-Rbp-1* alleles and homologues, our initial analysis shows a high degree of intraspecific amino acid variation encoded within the nematode populations examined. Evolutionary analysis suggested that a number of residues encoded by *Gp-Rbp-1* are under selective pressure. Previous analyses of genes encoding *G. rostochiensis* SPRYSEC proteins have shown that this gene family has undergone positive selection [19]. Whether *Gp*-RBP-1 is simply one member of a similarly expanded and diversified *G. pallida* SPRYSEC family remains to be elucidated. However, the *Gp*-RBP-1 sequences appear to be more similar to each other than to *Gm*-RBP-1 (Figure S1). As such, we suggest that the *Gp*-RBP-1 variants represent either different alleles of the same gene or the products of very recent duplications that can effectively be considered to be functionally the same. Thus, our analyses would indicate that the *Gp-Rbp-1* nematode parasitism gene has been subject to positive selection within nematode populations. It should be noted that sites under positive selection in *Gp*-RBP-1 were different than those identified in SPRYSEC homologs [19], although both analyses indicated selection on residues predicted to be at the surface of the protein in extended loops of the B30.2 domain (Figure 2). It has been suggested that the B30.2 domain in SPRYSEC proteins could provide a hypervariable binding surface which may be tuned to interact with a variety of protein partners [21]. For RBP-1 and SPRYSEC proteins this would presumably include plant protein targets including selection for interaction with virulence targets and/or selection for avoiding interactions with components involved in pathogen recognition. Such dual evolutionary forces may be further compounded by different selection pressures on alternate hosts and thus it may not be unexpected to find different positions under positive selection when comparing SPRYSEC and RBP-1 proteins.

Mutation and migration are two of the major evolutionary forces considered when assessing the risk of pathogen evolution in management of disease resistance and, due to their lifestyle, cyst nematodes have been associated with a low risk value for overcoming resistance [47]. However, both the high levels of gene flow shown to occur between populations [48],[49] and our finding of positive selection in the *Gp-Rbp-1* gene suggest that this risk may be higher than previously thought, with consequent implications for the development of durable resistance strategies.

High levels of variability have been shown for Avr determinants from two other eukaryotic pathogens, the ATR1 and ATR13 proteins from *H. parasitica*, and the AvrL456 proteins from *M. lini*, presumably because they are under selection pressure to evade the plant defense system [50],[51]. However, although ATR13 is highly variable, a single polymorphic amino acid determines recognition by RPP13, with a small number of other residues modulating the strength of this response [29]. This shows parallels to *Gp*-RBP-1, which also shows a great deal of variability (Figure 2), but whose recognition is ultimately determined by a single polymorphic residue (Figures 4 and 5). Thus, the *R* genes in question may not be a major factor in maintaining the diversity of these pathogen effectors. In particular, in the case of *Gp-RBP-1*, *Gpa2* does not restrict most European *G. pallida* populations, nor is it likely that *Gpa2* has exerted a significant pressure on nematode populations. Further PAML analyses using a data subset corresponding to sequences obtained from the four Peruvian *G. pallida* populations indicate that the polymorphism at position 187 in *Gp*-RBP-1 was under positive selection before *G. pallida* was introduced into Europe (data not shown). Thus the variability seen in *Gp-RBP-1* may be due to selection pressures exerted in the past within the native range of the pathogen, which may have included R proteins present in native hosts that recognize *Gp*-RBP-1. Alternatively, it has been proposed that *G. pallida* has adapted to new hosts on multiple occasions throughout its evolutionary history [52] and variation in *Gp*-RBP-1 may have been selected for during these adaptations. The role of RBP-1 and SPRYSEC proteins in parasitism is presently unknown. However, the *G. rostochiensis* protein SPRYSEC19 has been shown to interact physically with an NB-LRR protein without activating it, suggesting that it may play a role in inhibiting host defenses or that this family of proteins may be predisposed to recognition by NB-LRR proteins.

Like Rx, Gpa2 both binds to, and requires RanGAP2 for function (Figures 6 and S3). Given the specific interaction of RanGAP2 with Rx-like proteins and a lack of obvious signaling function, we have suggested that RanGAP2 may play a role in recognition by Gpa2 and Rx [15]. Indeed, multiple examples exist where proteins that bind to the N termini of NB-LRR proteins mediate recognition of Avr proteins, including the ternary interactions of AvrPto/Pto/Prf, AvrPphB/PBS1/RPS5, AvrRpm1/RIN4/RPS1, AvrRpt2/RIN4/RPS2, and p50/NRIP1/N [34],[53],[54],[55]. How can these observations be reconciled with domain swapping experiments demonstrating that the LRR domain determines recognition specificity [17],[30],[56],[57],[58]? The enhancement of Gpa2-mediated responses by tethering RanGAP2 to *Gp*-RBP-1 are consistent with a role for RanGAP2 as a recognition co-factor (Figure 7) that initially interacts with the Avr protein. However, tethering is not sufficient to induce activation of Gpa2 by non-recognized versions of *Gp*-RBP-1 nor is it sufficient to activate the Rx protein (Figures S4-S6). Thus, despite a prerequisite for an interaction with RanGAP2, it appears that the LRR domain determines which interactions will be productive. Such a scenario may explain apparently contradictory reports showing both direct and indirect interactions between the TIR-NB-LRR protein N and its cognate Avr determinant the p50 subunit of the tobacco mosaic virus (TMV) replicase. In the plant cell, P50 interacts with N only in the presence of the chloroplast protein NRIP1 [54], whereas there appears to be a direct interaction between N and p50 in the yeast two-hybrid system and *in vitro* [59]. A general mechanism for NB-LRR recognition of their cognate Avr determinants through a two-step process could reconcile such discrepancies. Indeed the N/p50 example would suggest that the NRIP1/TIR complex might stabilize a subsequent interaction between p50 and the N LRR domain. Furthermore, such a scenario could provide a mechanism to explain how NB-LRR proteins might evolve new recognition specificities without having to evolve to bind new cellular recognition co-factors. Further work will be required to determine whether such recognition co-factors are differentially modified by Avr proteins, resulting in activation of the NB-LRR, or whether they act to somehow present Avrs to the LRR domain which in turn mediates recognition. It is notable that although we initially tested *Gp*-RBP-1 due to its homology to the putative Ran-binding protein, RanBPM, there are legitimate doubts as to whether RanBPM actually binds Ran GTPase [21] and we are unable to co-immunoprecipitate Ran GTPase with *Gp*-RBP-1 (data not shown). Thus, it will be of interest to identify the virulence targets of RBP-1 proteins to determine whether RBP-1 proteins target RanGAP2 as predicted by the guard hypothesis [6] or whether RanGAP2 simply resembles the true virulence target(s) of RBP-1 as predicted by the decoy model [7].

## Materials and Methods

### Plant material and transient expression

*N. benthamiana* and *N. tabacum* plants were germinated and grown in a glass house or growth chambers maintained at 23°C. All experiments were repeated at least three times. Virus-induced gene silencing (VIGS), transient expression of proteins (Agro-expression), protein extraction, immuno-precipitation and immuno-blotting were carried out as previously described [15].

Transgenic *N. benthamiana* expressing *Gpa2* from the *Rx* native promoter were generated by stable transformation using *A. tumefaciens* strain LBA4404 carrying binary vector clone pB1-Gpa2 as previously described [15]. Transgenic *N. benthamiana* were generated to stably express RanGAP2 WPP:EGFP:HA and EGFP:HA from the cauliflower mosaic virus (CaMV) 35S promoter by transforming leaf tissue using *A. tumefaciens* strain C58C1 carrying binary vector constructs pBIN61-WPP:EGFP:HA or pBIN61-EGFP:HA (described below), and selecting on kanamycin. Transgenic *N. tabacum* expressing *Gpa2* from the *GPAII* native promoter were generated by stable transformation using *A. tumefaciens* strain pMOG101 carrying binary vector *pBIN+GPAII::Gpa2*.

### Plasmid construction

For generation of expression clones, all inserts were ligated into 5′ *Xba*I and 3′ *Bam*HI sites of the pBIN61 binary vector series unless otherwise indicated. This vector series contains epitope tags, or the enhanced red-shifted variant of jelly fish green fluorescent protein (EGFP) with an HA epitope tag, positioned for carboxy-terminal tagging of inserts in frame with the *Bam*HI site [17],[60],[61]. To obtain the complete *Gp-Rbp*-1 ORF, cDNA prepared from *G. pallida* pathotype (Pa) 2/3 population Chavornay [18] was amplified with primers GpaRBPMForSP (5′-CTCTAGATTTATTGCCCCCAAAATG-3′) and GpaRBPMstopRev (5′-GGATCCAGCAAACCCATCATAAATTCTCG-3′) and ligated into the pGEM-T vector. A pGEM-T clone was used to amplify fragments that 1) had the signal peptide deleted using primers GpaRBPMforXba (5′-CTCTAGACCATGGAGTCGCCAAAACCAAAC-3′) plus GpaRBPMstopRev; 2) had the stop codon changed to a *Bam*HI site for epitope tagging, using primers GpaRBPMForSP and GpaRBPMrevBam (5′-CCTGGATCCTAAATTCTCGTTTTTC-3′) or 3) had both the signal peptide deletion and the *Bam*HI site substitution for the stop codon, using primers GpaRBPMforXba and GpaRBPMrevBam. Nematodes from virulent (Rookmaker, Pa-3) and avirulent (D383 Pa-2) population of *Globodera pallida* (Pa-2/3) were hatched from eggs in the presence of potato root diffusate. Juveniles in the preparasitic stage (J2) were collected and used for RNA extraction followed by cDNA synthesis (Super Script III, Invitrogen). All additional *G. pallida* and *G. mexicana* RBP-1 clones were obtained by amplification with primers GpaRBPMrevBam plus either Chav6-7forXba (TGTCTAGAACCATGGAGTCGCCAAAACCAAAC), Gmex-1forXba (TGTCTAGAACCATGGAGTCGCCAAAAACAAAC), or Gmex-2forXba (TGTCTAGAACCATGGAGTCATCCAGTCCTGGCAATAC). A fragment without the signal peptide and with a BamHI substitution of the stop codon was amplified from cDNA prepared from *Globodera rostochiensis* pathotype Ro_1_ kindly provided by X. Wang, using primers GroRBPMforXba (5′-CTCTAGACCATGGATTCGCCGCCGCCAAAAAC-3′) and GroRBPMrevBam (5′-GGATCCAAATGGGCCAAAGTTCG-3′). YFP N- and C-terminal fragments were amplified by PCR using the enhanced yellow fluorescent protein (EYFP) from the pSAT vector series as a template [62] with primers BamFor-N-YFP (5′-GGATCCGGGATGGTGAGCAAGGG-3′) plus BglRev-N-YFP (5′-CAGATCTGTCCTCGATGTTGTGG-3′) for the N-terminal fragment and BamFor-C-YFP (5′-GGATCCATGGGCGGCAGCGTGCAG-3′) plus BglRev-C-YFP (5′-CAGATCTCTTGTACAGCTCGTCCATGC-3′) for the C-terminal fragment. Inserts cloned into pGEM-T were digested with *Bam*HI and *Bgl*II and ligated into the *Bam*HI site of pBIN61 constructs with either a FLAG:6His (FH) or HA tag [60], allowing subsequent cloning of candidate genes in frame with the epitope tagged YFP fragment using the 5? *Bam*HI site. Site directed mutants and domain swap constructs were generated based on extension overlap PCR. Primers were designed to change proline 187 to serine in Rook-1 and Chav-7, and the equivalent serine to proline in Rook-4 and Gmex-1, and to fuse aa 23-95 of Gmex-1 to aa 121-265 of Chav-7 (Figure S1). The Chav-7 deletion constructs were generated by PCR and correspond to fragments expressing residues 82-265 and 121-265 of Chav-7. A methionine and an alanine residue were added to N-terminal deletion constructs.

The GPAII:Gpa2 construct was assembled from the promoter region of the Gpa2 gene, the coding sequence, and the 3′-UTR. First, the 3′-UTR of *Gpa2* (274 bp) was amplified from pBINRGC2 [13] using the primers 5UTRkp (5′-TGGTACCTTCTGCAGCGAGTAGTTAAGGTGTTCTGAGGAC-3′) and 3UTRrev (5′-CTTAATTAACCCGGGAGATTGAGGACTCCCAAGAAAGG-3′). The amplicon was subcloned into the *KpnI* and *PacI* sites of pRAP-YFP. The *Gpa2* promoter region (GPAII; 2744 bp upstream of start codon, including the 5′-UTR) was subcloned into wthe *Asc*I and *Nco*I sites of the pRAP-3′UTR-YFP to generate pRAP-GPAII-3′UTR-YFP. The 5′-end of the *Gpa2* coding sequence as PCR amplified from pBINRGC2 [13] using primers 5′GpRxbn (5′-TTTTTGGATCCATGGCTTATGCTGCTGTTACTTCCC-3′) and GpRxStuRev (5′-CAAAGAAAGAAGGCCTAGGAGTAC-3′). The *Nco*I and *Pst*I fragment was ligated together with an *Avr*II-*Pst*I fragment from pBINRGC2 into the *Nco*I and *Pst*I sites of pUCAP making pUCAP-Gpa2 [63]. The *Nco*I and *Pst*I fragment from the pUCAP-Gpa2 plasmid was subsequently into the *Nco*I and *Pst*I sites of pRAP-GPAII-3′UTR-YFP, resulting in pRAP-pGPAII::Gpa2-3′UTR-YFP. As a final cloning step, the *Asc*I-*Pac*I fragment of pRAP-pGPAII::Gpa2-3′UTR-YFP was ligated into corresponding sites in the binary plasmid pBIN+ resulting in pBIN-GPAII::Gpa2.

### DNA and protein sequences and analysis

DNA sequences were translated to protein and aligned using the Translator and ClustalW-based Aligner programs of the JustBio suite (Pierre Rodrigues, www.justbio.com/tools.php). New *Gp-Rpb-1* sequences functionally analyzed in this study have been deposited to GenBank/EMBL databases under the following accession numbers: AM491352 (Chav-1), AM491353 (Chav-2), AM491354 (Chav-3), AM491355 (Chav-4), AM491356 (Chav-5), FJ392678 (Chav-6), FJ392677 (Chav-7), EF423897 (Rook-1), EF423898 (Rook-2), EF423899 (Rook-3), EF423900 (Rook-4), EF4238901 (Rook-5), EF4238902 (Rook-6), EF423893 (D383-1), EF423894 (D383-2), EF423895 (D383-3), EF423896 (D383-4). New *G. mexicana Rbp-1* sequences analyzed in this study have been deposited to GenBank/EMBL databases under the following accession numbers: FJ392679 (Gmex-1), and FJ392680 (Gmex-2). Additional *G. pallida* sequences used for PAML analysis were: EU982195 (Luffness; GPE1), EU982196 (Ouessant; GPE2), EU982197 (Chavornay; GPE3), EU982198 (Duddingston; GPE5) and EU982199 (Guiclan; GPE6) from Europe; and EU982200 (Colque-cachi; GPS3), EU982201 Chamancalla; GPS5), EU982202 (Ballo-ballo; GPS7), EU982203 (Chocon; GPS8), EU982204 (Otuzco; GPS9), and EU982205 (Huamacucho; GPS10) from Peru. Additional sequences relevant for this report can be retrieved from the GenBank/EMBL databases under the following accession numbers: AJ251757 (*Gr-*RBP-1) AJ011801 (Rx), AJ249449 (Rx2), AJ249449 (Gpa2), AF172259 (PVX-CP), AF202179 (Bs2), and AM411448 (RanGAP2).

### Construction of the sequence data sets

Complementary DNAs encoding *Gp-Rbp1* were amplified from 13 *G. pallida* populations (7 European and 6 Peruvian) as described, using specific primers 5′IC5.2 and 3′IC5.2 [18]. The PCR products were cloned and sent to Macrogen (http://dna.macrogen.com) for sequencing. Multalin (http://bioinfo.genopole-toulouse.prd.fr/multalin) with DNA 5-0 alignment parameters was used for multiple sequence alignment [64]. The alignment was manually corrected when necessary. The MEGA program v 3.1 was used to obtain Neighbour-Joining trees [65].

### Evolutionary analysis: Identification of sites under positive selection

Selective pressures on RBP-1 sequences were evaluated using the ratio of nonsynonymous to synonymous substitution rates per site (ω = K_a_/K_s_) using the phylogenetic analysis by maximum likelihood (PAML), single-likelihood ancestor counting (SLAC), fixed-effects likelihood (FEL), internal branches fixed-effects likelihood (IFEL) and random effect likelihood (REL) methods implemented in the PAML package version 3.14 [66] or in the HYPHY package [67]. A value of ω = 1 reflects neutrality, ω<1 indicates purifying selection and ω>1 indicates positive selection. PAML analyses were done with the CODEML program (M1 vs M2 and M7 vs M8 models). The Bayes Empirical Bayes approach was used to calculate the posterior probabilities that each site fell into a different K_a_/K_s_ (or ω) class [68]. PAML assigns a likelihood score to models for selection. A likelihood score for a model incorporating positive selection that is higher than that for a null model without positive selection is evidence for positive selection. The significance of the differences was estimated by comparing the null model and positive selection model (2Δl) with a chi square table (Likelihood Ratio Test, LRT).

## Supporting Information

Figure S1 Sequence alignment of deduced RBP-1 proteins. Individual cDNA clones were obtained by RT-PCR of mRNA from larvae belonging to imported (Chavornay [CH], Rookmaker [NL], D383 [NL], Guiclan [FR] and Pukekohe [NZ]) and native (GPS4, GPS7, GPS9 and GPS10) *G. pallida* populations. Two sequences from *G. mexicana* (Gmex) were also included. Sequence alignment was generated using Multalin software. High consensus amino acids are coloured in red, low consensus amino acids are coloured in blue. Sequence regions not considered in the PAML and other evolutionary analyses are indicated by asterisks above the alignment. Positions of the six *Gp-Rbp1* introns are indicated by triangles and the repeated PRY domains are indicated by a solid line above the alignment.
(2.75 MB TIF)

Figure S2 Interaction between RanGAP2 and Gpa2 through their amino-terminal domains. (A) FLAG-tagged CC domains from Gpa2 and Bs2 were transiently co-expressed by agro-infiltration with RanGAP2 or fragments thereof as EGFP:HA fusion proteins in *N. benthamiana*. Reciprocal co-immunoprecipitations with anti-FLAG and anti-HA conjugated agarose beads demonstrate that the RanGAP2 amino-terminal WPP domain interacts specifically with the Gpa2 CC domain when analyzed on immunoblots detecting the epitope tags. (B) A dominant-negative version of RanGAP2, consisting of a 133 amino acid fragment from the RanGAP2 amino terminus was expressed transgenically as a GFP fusion protein in *N. benthamiana* (WPP:EGFP:HA). Control lines were also generated expressing EGFP:HA protein. Leaves were infiltrated with 35S::Pto plus 35S::AvrPto or pB1-Gpa2 plus pBin61-EGFP:HA as positive and negative HR controls, respectively. The RanGAP2 dominant-negative effect was assayed by co-infiltration of pB1-Rx:HA with pBin61-CP, or pB1-Gpa2 with pBin61-*Gp*-RBP-1:EGFP:HA.
(2.93 MB PDF)

Figure S3 Enhancement of HR through Gpa2 by complementing YFP fragments fused to RanGAP2 and *Gp*-RBP-1 is specific for avirulent variants of *Gp*-RBP-1. Reciprocal YFP fragment fusions of *Gp*-RBP-1 (Rook-4 and Rook-6) were co-expressed in *Gpa2*-transgenic tobacco together with the indicated nYF and cYF fusions of RanGAP2 and GUS (A-C). Complementing pairs of YFP fragment fusion proteins are noted in yellow, and non-complementing combinations in white. Note that Rook-6:nYF induces a weaker response than Rook-6:cYF (A), similar to that seen with D383-2:nYF (Figure 7A). (D) HR enhancement did not result simply from the co-expression of D383-2 with RanGAP2:nYF, RanGAP2:cYF or RanGAP2:F demonstrating a requirement for YFP complementation in the HR enhancement.
(3.65 MB PDF)

Figure S4 Enhancement of Gpa2-mediated HR by YFP complementation correlates with physical interaction between RanGAP2 and *Gp*-RBP-1 fusion proteins. In order to demonstrate physical interaction between YFP fragment fusions, the FLAG epitope tag of nYF and cYF fusions was replaced with an HA epitope tag (nYHA and cYHA). Rook-4, Rook-6 and GUS fusions with either nYHA, cYHA, nYF or cYF were transiently expressed in Gpa2-transgenic tobacco either alone (right hand side) or together with either RanGAP2:cYHA, RanGAP2:cYF or RanGAP2:nYF (A). HR induction results with HA fusions were similar to those obtained in experiments in which all fusions were tagged with the FLAG-epitope (compare top versus bottom panels and this figure to Figure S3). (B) Similar combinations of YFP fusion proteins were co-expressed in wild-type *N. benthamiana*. Protein extracts were subjected to-immunopreciptation (IP) was performed with anti-FLAG agarose beads followed by immunoblotting (IB) with anti-FLAG and anti-HA antisera. Anti-HA immunoprecipitation followed by anti-HA immunoblotting was also performed to detect HA epitope-tagged fusions for confirmation of expression levels. Detection of co-immunoprecipitated proteins shows that only combinations with complementing YFP fragments interact.
(2.95 MB PDF)

Figure S5 Requirement for NB-LRR specificity determination for HR elicitation by YFP complemented *Gp*-RBP-1. The indicated combinations of YFP fragment fusion proteins were transiently expressed by agro-infiltration in Rx-transgenic tobacco leaves as in Figure S4A. A lack of HR is indicated by (-).
(0.04 MB PDF)

Table S1 Evolutionary analysis of RBP-1 sequence dataset. (A) PAML analyses were carried out using the codeml module of PAML. “*p*” is the number of parameters in the ω distribution. “*l*” corresponds to the log-likelihood value. Positive selection sites with posterior probability >95% are indicated in red. LRT = Likelihood ratio test in which gap between log-likelihood values (2Δl) were compared to a chi-square table of critical values with 2 df (results shown under the *P* indication). (B) Positively selected sites in RBP-1 sequence dataset identified by PAML and at least one other method. Substitution rates per site (ω = K_a_/K_s_) were evaluated using the single-likelihood ancestor counting (SLAC), fixed-effects likelihood (FEL), internal branches fixed-effects likelihood (IFEL) and random effect likelihood (REL) methods. For the SLAC and FEL methods, the numbers in parentheses refer to the obtained *P* values for the appropriate position. For the REL and PAML methods, the numbers in parentheses refer to the posterior probabilities of the Bayes Empirical Bayes (BEB) analysis. Positive selection sites detected significantly by each test are highlighted in bold.
(0.09 MB PDF)
