# Supplementary figures and images for: The Cyst Nematode SPRYSEC Protein RBP-1 Elicits Gpa2- and RanGAP2-Dependent Plant Cell Death
Source: PLoS Pathog. 2009 Aug 28;5(8):e1000564. doi: 10.1371/journal.ppat.1000564 (PMC2727447; doi:10.1371/journal.ppat.1000564)

A

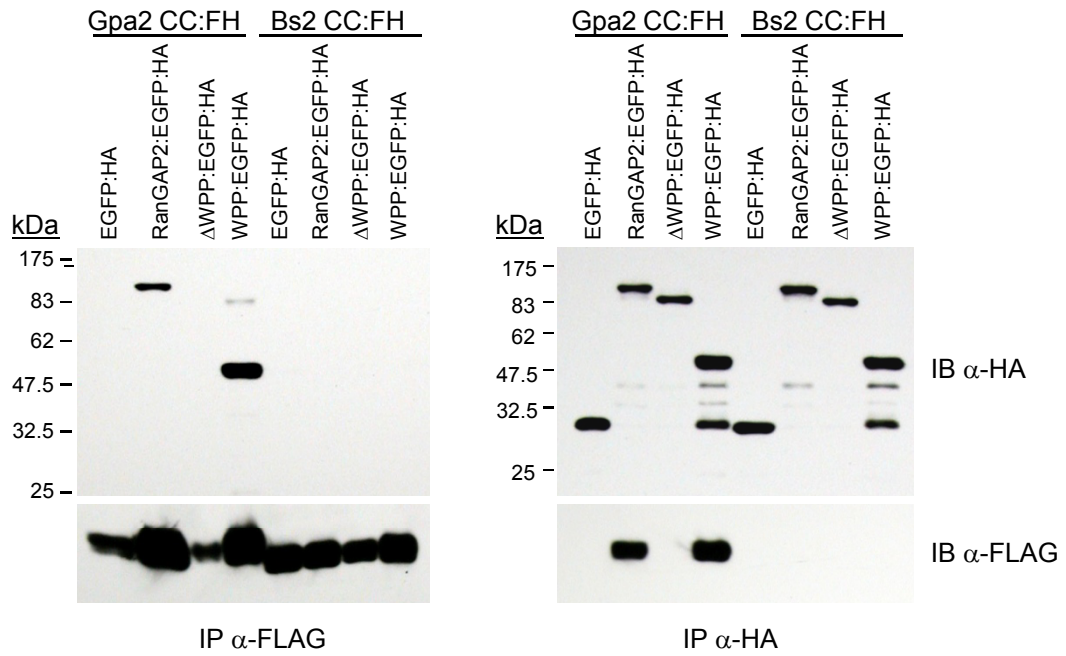

B

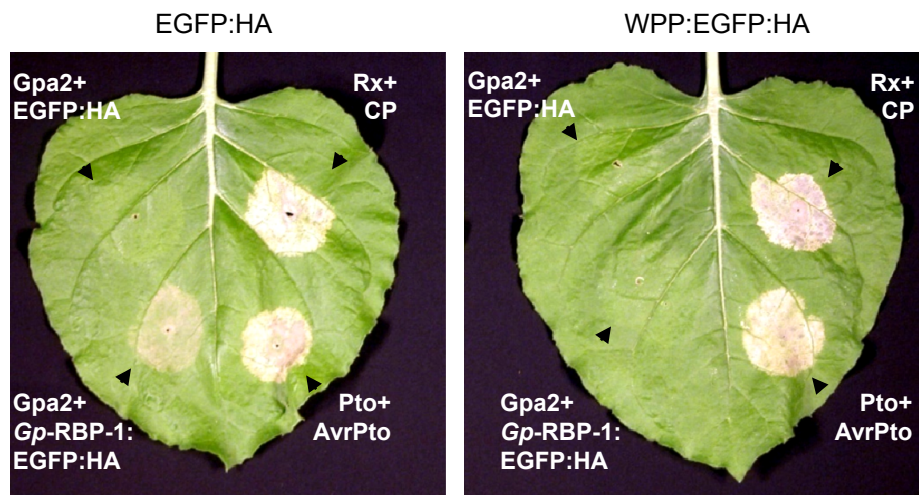

Supplement: Figure S2 — Interaction between RanGAP2 and Gpa2 through their amino-terminal domains. (A) FLAG-tagged CC domains from Gpa2 and Bs2 were transiently co-expressed by agro-infiltration with RanGAP2 or fragments thereof as EGFP:HA fusion proteins in N. benthamiana. Reciprocal co-immunoprecipitations with anti-FLAG and anti-HA conjugated agarose beads demonstrate that the RanGAP2 amino-terminal WPP domain interacts specifically with the Gpa2 CC domain when analyzed on immunoblots detecting the epitope tags. (B) A dominant-negative version of RanGAP2, consisting of a 133 amino acid fragment from the RanGAP2 amino terminus was expressed transgenically as a GFP fusion protein in N. benthamiana (WPP:EGFP:HA). Control lines were also generated expressing EGFP:HA protein. Leaves were infiltrated with 35S::Pto plus 35S::AvrPto or pB1-Gpa2 plus pBin61-EGFP:HA as positive and negative HR controls, respectively. The RanGAP2 dominant-negative effect was assayed by co-infiltration of pB1-Rx:HA with pBin61-CP, or pB1-Gpa2 with pBin61-Gp-RBP-1:EGFP:HA. (2.93 MB PDF) [file ppat.1000564.s002.pdf]

A

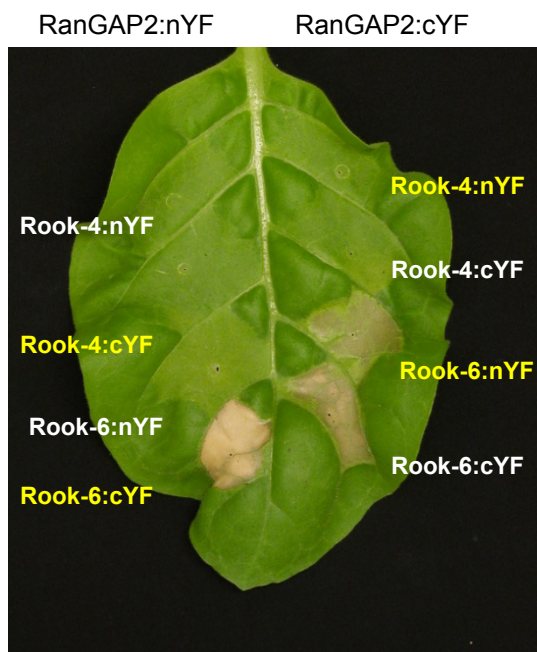

B

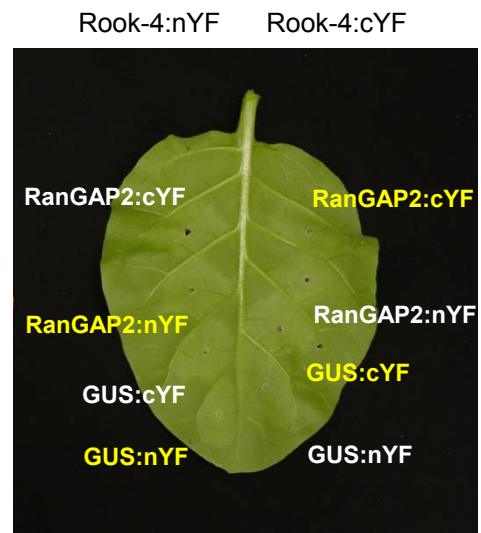

C

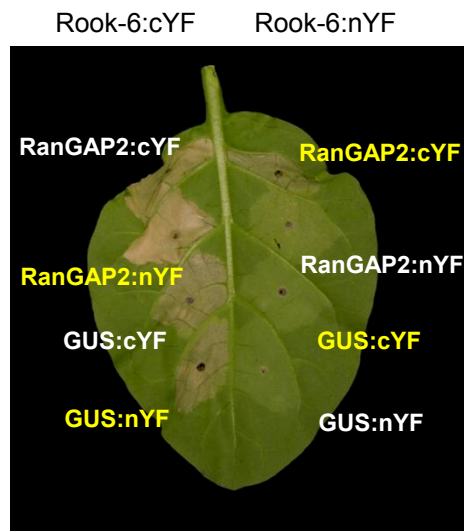

D

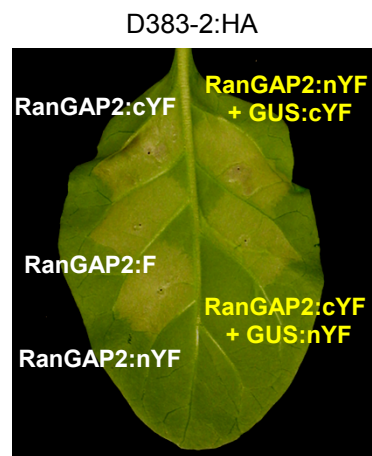

Supplement: Figure S3 — Enhancement of HR through Gpa2 by complementing YFP fragments fused to RanGAP2 and Gp-RBP-1 is specific for avirulent variants of Gp-RBP-1. Reciprocal YFP fragment fusions of Gp-RBP-1 (Rook-4 and Rook-6) were co-expressed in Gpa2-transgenic tobacco together with the indicated nYF and cYF fusions of RanGAP2 and GUS (A-C). Complementing pairs of YFP fragment fusion proteins are noted in yellow, and non-complementing combinations in white. Note that Rook-6:nYF induces a weaker response than Rook-6:cYF (A), similar to that seen with D383-2:nYF (Figure 7A). (D) HR enhancement did not result simply from the co-expression of D383-2 with RanGAP2:nYF, RanGAP2:cYF or RanGAP2:F demonstrating a requirement for YFP complementation in the HR enhancement. (3.65 MB PDF) [file ppat.1000564.s003.pdf]

A

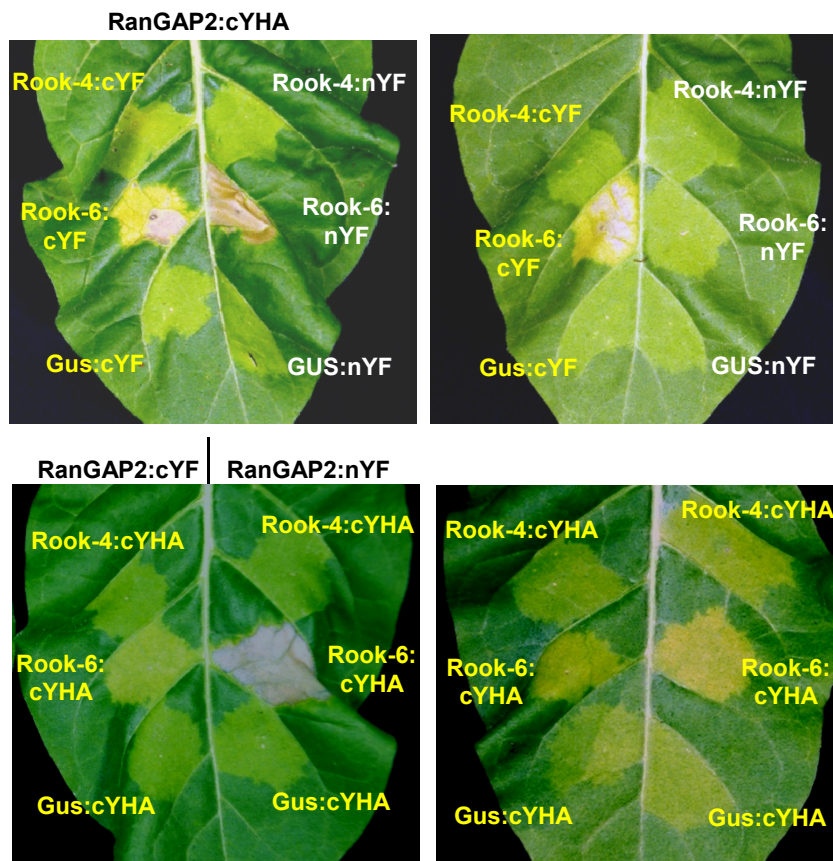

B

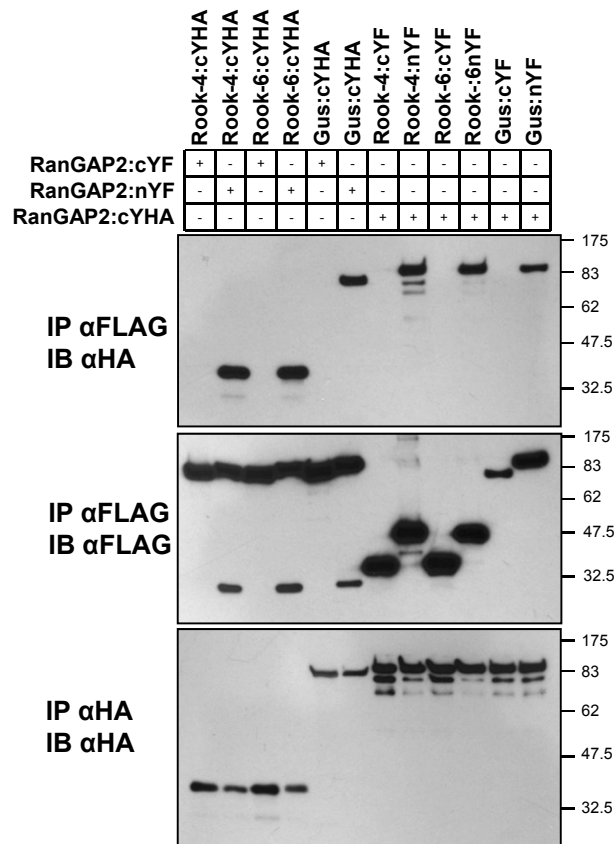

Supplement: Figure S4 — Enhancement of Gpa2-mediated HR by YFP complementation correlates with physical interaction between RanGAP2 and Gp-RBP-1 fusion proteins. In order to demonstrate physical interaction between YFP fragment fusions, the FLAG epitope tag of nYF and cYF fusions was replaced with an HA epitope tag (nYHA and cYHA). Rook-4, Rook-6 and GUS fusions with either nYHA, cYHA, nYF or cYF were transiently expressed in Gpa2-transgenic tobacco either alone (right hand side) or together with either RanGAP2:cYHA, RanGAP2:cYF or RanGAP2:nYF (A). HR induction results with HA fusions were similar to those obtained in experiments in which all fusions were tagged with the FLAG-epitope (compare top versus bottom panels and this figure to Figure S3). (B) Similar combinations of YFP fusion proteins were co-expressed in wild-type N. benthamiana. Protein extracts were subjected to-immunopreciptation (IP) was performed with anti-FLAG agarose beads followed by immunoblotting (IB) with anti-FLAG and anti-HA antisera. Anti-HA immunoprecipitation followed by anti-HA immunoblotting was also performed to detect HA epitope-tagged fusions for confirmation of expression levels. Detection of co-immunoprecipitated proteins shows that only combinations with complementing YFP fragments interact. (2.95 MB PDF) [file ppat.1000564.s004.pdf]
