# Supplementary material for: The Cyst Nematode SPRYSEC Protein RBP-1 Elicits Gpa2- and RanGAP2-Dependent Plant Cell Death
Source: PLoS Pathog. 2009 Aug 28;5(8):e1000564. doi: 10.1371/journal.ppat.1000564 (PMC2727447; doi:10.1371/journal.ppat.1000564)
Supplement: Figure S5 — Requirement for NB-LRR specificity determination for HR elicitation by YFP complemented Gp-RBP-1. The indicated combinations of YFP fragment fusion proteins were transiently expressed by agro-infiltration in Rx-transgenic tobacco leaves as in Figure S4A. A lack of HR is indicated by (-). (0.04 MB PDF) [file ppat.1000564.s005.pdf]

Rx-transgenic Tobacco

	Rook4: cYF	Rook6: cYF	Gus: cYF
RanGAP2:cYHA	-	-	-
	-	-	-
	Rook4: nYF	Rook6: nYF	Gus: nYF
