# Supplementary material for: The Cyst Nematode SPRYSEC Protein RBP-1 Elicits Gpa2- and RanGAP2-Dependent Plant Cell Death
Source: PLoS Pathog. 2009 Aug 28;5(8):e1000564. doi: 10.1371/journal.ppat.1000564 (PMC2727447; doi:10.1371/journal.ppat.1000564)
Supplement: Table S1 — Evolutionary analysis of RBP-1 sequence dataset. (A) PAML analyses were carried out using the codeml module of PAML. “p” is the number of parameters in the ω distribution. “l” corresponds to the log-likelihood value. Positive selection sites with posterior probability >95% are indicated in red. LRT = Likelihood ratio test in which gap between log-likelihood values (2Δl) were compared to a chi-square table of critical values with 2 df (results shown under the P indication). (B) Positively selected sites in RBP-1 sequence dataset identified by PAML and at least one other method. Substitution rates per site (ω = Ka/Ks) were evaluated using the single-likelihood ancestor counting (SLAC), fixed-effects likelihood (FEL), internal branches fixed-effects likelihood (IFEL) and random effect likelihood (REL) methods. For the SLAC and FEL methods, the numbers in parentheses refer to the obtained P values for the appropriate position. For the REL and PAML methods, the numbers in parentheses refer to the posterior probabilities of the Bayes Empirical Bayes (BEB) analysis. Positive selection sites detected significantly by each test are highlighted in bold. (0.09 MB PDF) [file ppat.1000564.s006.pdf]

A

Model Code	*p*	*l*	positively selected sites	Likelihood ratio test (LRT)			
M0 (one ratio)	1	-2102.95	none	LRT	2Δl	df	*P*
M1a (neutral)	1	-2094.06	none	M1-M2	30.3	2	< 0.001 significant
M2a (positive selection)	3	-2078.90	23A, 59T, 84T, 100Y, 102K, 103A, 106G, 174G, 187P, 203N				
M7 (beta)	2	-2094.24	none	M7-M8	30.9	2	< 0.001 significant
M8 (beta & ω)	4	-2078.76	23A, 59T, 84T, 88L, 100Y, 101T, 102K, 103A, 106G, 119K, 128V, 174G, 187P, 202E, 203N				

B

**PAML position**	**Counting method**	**Likelihood methods**		
	**SLAC**	**FEL**	**REL**	**PAML**
59 T	6.913 (0.32)	5.463 (0.11)	**2.744 (0.99)**	**5.651 (0.92)**
119 K	7.100 (0.41)	5.415 (0.17)	**2.679 (0.99)**	3.344 (0.54)
174 G	7.390 (0.27)	5.379 (0.13)	**2.687 (0.99)**	4.580 (0.75)
187 P	**13.578 (0.09)**	**12.024 (0.02)**	**9.645 (0.99)**	**6.117 (0.99)**
